# Transcutaneous versus arterial carbon dioxide monitoring in adult polysomnography studies

**DOI:** 10.1093/sleep/zsaf277

**Published:** 2025-09-13

**Authors:** Keeley A Miller, Thomas J Churchward, Julie Tolson, Warren R Ruehland, Christine F McDonald, Christopher J Worsnop

**Affiliations:** Department of Medicine, University of Melbourne, Melbourne, VIC, Australia; Department of Respiratory and Sleep Medicine, Austin Health, Heidelberg, VIC, Australia; Institute for Breathing and Sleep, Heidelberg, VIC, Australia; Department of Medicine, University of Melbourne, Melbourne, VIC, Australia; Department of Respiratory and Sleep Medicine, Austin Health, Heidelberg, VIC, Australia; Institute for Breathing and Sleep, Heidelberg, VIC, Australia; Department of Respiratory and Sleep Medicine, Austin Health, Heidelberg, VIC, Australia; Institute for Breathing and Sleep, Heidelberg, VIC, Australia; Department of Medicine, University of Melbourne, Melbourne, VIC, Australia; Department of Respiratory and Sleep Medicine, Austin Health, Heidelberg, VIC, Australia; Institute for Breathing and Sleep, Heidelberg, VIC, Australia; Department of Medicine, University of Melbourne, Melbourne, VIC, Australia; Department of Respiratory and Sleep Medicine, Austin Health, Heidelberg, VIC, Australia; Institute for Breathing and Sleep, Heidelberg, VIC, Australia

**Keywords:** carbon dioxide, hypercapnia, transcutaneous blood gas monitoring, arterial blood gas, polysomnography, respiratory failure

## Abstract

**Study objectives:**

To determine the accuracy of transcutaneous carbon dioxide measurements using SenTec tcPCO_2_ during adult polysomnography (PSG) compared to arterial PCO_2_.

**Methods:**

In consecutive patients having PSG with transcutaneous CO_2_ (tcPCO_2_) monitoring using a SenTec monitor, arterial blood gas samples were taken at the beginning and at the end of each sleep study. tcPCO_2_ measurements recorded at 0, 60, and 120 s after arterial sampling was determined in 51 participants who underwent PSG with tcPCO_2_ monitoring at the Austin Health Sleep Laboratory (Heidelberg, VIC, Australia) during the period 01/05/22–10/06/22. The mean of differences between arterial carbon dioxide tension (P_a_CO_2_) and the predicted value based on tcPCO_2_ measurements determined at each time point for evening and morning arterial samples.

**Results:**

Data were obtained from 37 participants. A statistically significant difference in the tcPCO_2_ values at the point of arterial sampling was found, with tcPCO_2_ on average 2.2–2.8 mmHg less than P_a_CO_2_ at various timepoints after arterial sampling. The upper limit of measurement accuracy represented a moderate difference (2–4.9 mmHg), within the clinically acceptable range of −7.5 to +7.5 mmHg variation from P_a_CO_2_ values.

**Conclusions:**

The SenTec tcPCO_2_ electrode was found to be an appropriate alternative to arterial sampling for assessment of arterial carbon dioxide in adult patients in the PSG setting, with no significant difference in tcPCO_2_ accuracy based on the timing of transcutaneous measurements (up to 2 min) following arterial sampling.

## Teaser

**Statement of Significance** Given sleep-disordered hypoventilation and hypercapnic respiratory failure can be effectively managed by non-invasive positive pressure ventilation, accurate measurement of arterial CO_2_ in this setting is crucial. Previous studies have addressed tcPCO_2_ effectiveness across critical care and surgical settings, with varying results. The significance of this study is addressing the validity of tcPCO_2_ technology specifically within the polysomnography setting, which services a vastly different patient population to critical care settings. Patients undergoing sleep studies are typically well and present for the purpose of diagnosing/monitoring sleep-disordered breathing. This study demonstrates how tcPCO_2_ can be effectively used in place of arterial sampling to identify hypercapnia in such patients, where it may not be acceptable to expose patients to the risks associated with invasive sampling.

## Graphical Abstract

**Graphical Abstract:**
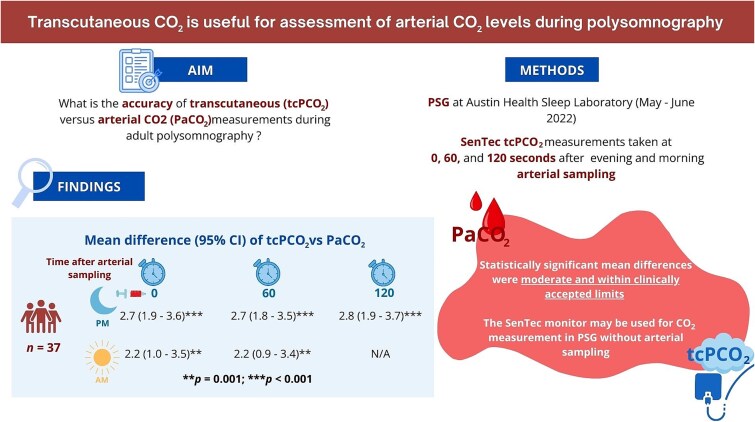

## Introduction

Maintenance of carbon dioxide (CO_2_) levels relies upon maintaining the balance between the metabolic production of CO_2_ and its elimination via alveolar ventilation [1, 2]. Hypercapnic respiratory failure can arise acutely or chronically when arterial carbon dioxide tension (PaCO_2_) exceeds 45 mmHg [3–5]. This can arise in an array of respiratory conditions including exacerbations of chronic obstructive pulmonary disease (COPD), asthma, and cardiogenic pulmonary edema [1], in addition to obesity hypoventilation syndrome (OHS) [6], chest wall deformity, and neuromuscular diseases [3, 4, 7]. As sleep is associated with changes in muscle activity, airway resistance, and central control of respiration [8], hypoventilation is more likely to result during sleep and can lead to hypercapnia and chronic respiratory failure [9], requiring nocturnal breathing support.

Non-invasive positive pressure ventilation (NIPPV) is widely accepted as the standard treatment for chronic hypercapnic respiratory failure, correcting both hypoventilation and the resulting hypercapnia [1, 4]. Use of nocturnal NIPPV has been associated with reductions in mortality, need for intubation, and fewer hospital admissions in patients with COPD; reduced mortality and a better quality of life in patients with neuromuscular disease; and improved sleep quality in those with OHS [4]. Reported NIPPV initiation criteria are variable, but most include hypercapnia (PaCO_2_ > 45 mmHg) as either a single criterion or part of combination criteria which may also include hypoxia, arterial blood pH, or pulmonary function results [4, 7]. As such, it is important to appropriately identify hypercapnic individuals who are likely to benefit from NIPPV therapy, which relies upon the accurate measurement of arterial CO_2_ levels.

Arterial blood gas (ABG) analysis is the gold standard for measuring arterial CO_2_ levels; however, this invasive test is limited by only providing a point P_a_CO_2_ measurement at the time the sample was taken, and the risks of pain, hemorrhage, and infection from the procedure [2, 5]. Other non-invasive methods of estimating P_a_CO_2_ have been studied, namely transcutaneous carbon dioxide (tcPCO_2_) sensors and end-tidal CO_2_ monitoring [2, 7], however variability in reported accuracy has restricted widespread clinical use [10]. tcPCO_2_ is of particular interest for use in polysomnography (PSG) studies as a way of detecting temporal trends in CO_2_ levels to identify hypercapnic patients who are likely to benefit from NIPPV.

There are two models of tcPCO_2_ currently available in Australia, SenTec (SenTec AG, Therwil, Switzerland) tcPCO_2,_ and Radiometer (Radiometer Medical ApS, Brønshøj, Denmark) TCM5 FLEX with TOSCA electrode. These sensors are based on the Stow-Severinghaus electrode [5, 11, 12], which locally heats the skin to vasodilate blood vessels and facilitate CO_2_ diffusion through a CO_2_ permeable membrane into the electrode and compares pH change to their in-built reference electrode [12,13]. CO_2_ tension just below the skin is typically greater than CO_2_ tension in arterial blood, thus these electrodes utilize “The Severinghaus equation” algorithm to correct CO_2_ tension values to 37.0°C and approximate arterial CO_2_ [10, 12].

Numerous studies have looked at the accuracy of tcPCO_2_ measurements compared to PaCO_2_ from ABG analysis in various clinical settings [10], with varying results. Most studies utilized ±7.5 mmHg (or 1 kPa) as the clinically acceptable limit of agreement, based on a clinical definition recommended by the American Association for Respiratory Care therapists [10, 14]. A number of studies have found the difference between PaCO_2_ and tcPCO_2_ to exceed acceptable population limits of agreement in various clinical settings [5, 15, 16], while others have found the difference between PaCO_2_ and tcPCO_2_ to fall within the clinically acceptable limits [14, 17]. A recent systematic review investigating a range of tcPCO_2_ monitors reported only small bias between PaCO_2_ and tcPCO_2_ measures (0.1 mmHg), however noted significant variation between measures in these patients (−15 mmHg to +15 mmHg) [10], exceeding the clinically accepted limit of agreement. Through subgroup meta-analyses, that study identified the site, temperature, and type of tcPCO_2_ electrode as factors that influenced tcPCO_2_ accuracy.

The primary objective of our study was to determine the accuracy of transcutaneous CO_2_ measurement using SenTec tcPCO_2_ during adult PSG studies. Given the inherent delay in CO_2_ changes occurring in the arteries being detected transcutaneously, a subsequent objective aimed to investigate the impact of tcPCO_2_ measurement stability/drift over time.

## Materials and Methods

The subjects were consecutive patients having PSG with tcPCO_2_ monitoring for either the investigation or the management of chronic respiratory failure at the Austin Health Sleep Laboratory (Heidelberg, VIC, Australia) during the period 1/05/2022 to 10/06/2022, inclusive. PSG studies without successful ABG samples or missing tcPCO_2_ data were excluded.

The following information was extracted from each participant’s data: study date, PaCO_2_ from two ABGs (one evening, one the following morning), the conditions at the time of ABG sample collection, six tcPCO_2_ measurements (obtained at 0, 60, and 120 seconds after evening and morning arterial sampling), electrode temperature, location of electrode placement, patient age, sex, height, weight, body mass index (BMI), apnea–hypopnea index, the reason for the PSG study and if treatment was used during the PSG, the mode that was used. Three time points were used to note the tcPCO_2_ readings as in clinical practice there can be a delay while the ABG sample is being taken.

The accuracy of the SenTec sensor was assessed by comparing the difference in CO_2_ measures obtained by ABG and tcPCO_2_. Statistical differences were assessed using a two-tailed 95% confidence interval (CI) for the mean difference in CO_2_ measurement determined by paired *t*-tests in spss27 statistical software. Clinical accuracy of tcPCO_2_ monitoring was judged based on mean CO_2_ differences within the accepted ±7.5 mmHg range (as used in previous research) [10, 14]. This difference was then defined to be marginal (0–1.9 mmHg), moderate (2–4.9 mmHg), or substantial (≥ 5 mmHg).

tcPCO_2_ measurement stability was assessed by considering variation in tcPCO_2_ values at 60 and 120 seconds after ABG sample collection, analyzed by the same statistical methods described above.

The pooled standard deviation of the SenTec tcPCO_2_ device was determined to be 4.6 mmHg [10]. Given this standard deviation and the following parameters, *α* = 0.05, power = 0.8, and delta = 7.5, a sample size of 36 participants was required to ensure adequate data to answer the prescribed research question of this superiority study.

The Austin Health Human Research Ethics Committee (HREC) approved this research. Project HREC number: HREC/87286/Austin-2022.

## Results

Data from 51 consecutive PSG patients with tcPCO_2_ monitoring were considered, with data from 13 participants excluded due to the absence of ABG data and a further one participant excluded due to missing tcPCO_2_ data. The characteristics of the included participants are shown in Table 1. Electrode location was not recorded for almost half of the included participants (*n* = 18), with the remaining participants having the electrode placed on either their forehead (*n* = 10) or chest (*n* = 9) throughout the study. As a result, it was not possible to determine the impact of electrode location on tcPCO_2_ accuracy.

**Table 1:** Characteristics of study participants

Characteristic	All participants
Age (*n* = 37)	
*M* ± *SD* (years)	57.6 ± 13.9
Range (years)	19–78
Sex (*n* = 37)	
Female (*n*, %)	17 (45.9%)
Male (*n*, %)	20 (54.1%)
BMI (*n* = 30)	
*M* ± *SD*	37.8 ± 13.0
BMI ≤ 18 (*n*, %)	0 (0.0%)
18 < BMI ≤ 25 (*n*, %)	3 (8.1%)
25 < BMI ≤ 30 (*n*, %)	5 (13.5%)
30 < BMI ≤ 40 (*n*, %)	13 (35.1%)
BMI > 40 (*n*, %)	9 (24.3%)
PSG type (*n* = 37)	
Diagnostic (*n*, %)	8 (21.6%)
Treatment (*n*, %)	29 (78.4%)
Reason for PSG study (*n* = 37)	
Hypoventilation investigation (*n*, %)	6 (16.2%)
CPAP titration (*n*, %)	11 (29.7%)
NIPPV setting titration (*n*, %)	18 (48.6%)
Diaphragm dysfunction investigation (*n*, %)	2 (5.4%)
AHI (*n* = 7)	
*M* ± *SD*	39.4 ± 32.2
Range	1.4–93.6
Treatment mode (*n* = 37)	
None (*n*, %)	9 (24.3%)
CPAP (*n*, %)	11 (29.7%)
NIPPV (*n*, %)	17 (45.9%)

Demographics of participants including number (*n*), percentage of sample size, mean (*M*), and standard deviation (*SD*). BMI: body mass index; PSG: polysomnography; CPAP: continuous positive airway pressure; NIPPV: non-invasive positive pressure ventilation; AHI: apnea–hypopnea index; tcPCO_2_: transcutaneous carbon dioxide monitoring.

The comparisons between the tcPCO_2_ and PaCO_2_ measurements are shown in Table 2. Of the 37 participants included in this study, 34 had a successful evening ABG with a mean of difference between P_a_CO_2_ and tcPCO_2_ of 2.7 mmHg (*p* < .001) at 0 s, 2.7 mmHg (*p* < .001) at 60 s, and 2.8 mmHg (*p* < .001) at 120 s. Thirty two participants had a successful morning ABG performed with a mean difference of 2.2 mmHg (*p* = .001) at 0 s and 2.2 mmHg (*p* = .001) at 60 s. Data for the tcPCO_2_ measurement at 120 s did not meet assumptive criteria for a paired *t*-test; thus, a nonparametric sign test was performed (*p* = .001; Table 3).

**Table 2:** Results of morning and evening CO_2_  *t*-test analysis

Statistic	PM (*n* = 34)	AM (*n* = 32)
tcPCO_2_ (0)		
Mean tcPCO_2_	43.2 mmHg	43.8 mmHg
Mean PaCO_2_	45.9 mmHg	46.0 mmHg
Mean difference	2.7 mmHg (*p* < 0.001)	2.2 mmHg (*p* = .001)
95% CI	1.9–3.6 mmHg	1.0–3.5 mmHg
tcPCO_2_ (60)		
Mean tcPCO_2_	43.3 mmHg	43.8 mmHg
Mean PaCO_2_	46.0 mmHg	46.0 mmHg
Mean difference	2.7 mmHg (*p* < .001)	2.2 mmHg (*p* = .001)
95% CI	1.8–3.5 mmHg	0.9–3.4 mmHg
tcPCO_2_ (120)		*n* = 29
Mean tcPCO_2_	43.1 mmHg	42.9 mmHg
Mean PaCO_2_	45.9 mmHg	46.0 mmHg
Mean of differences	2.8 mmHg (*p* < 0.001)	NA
95% CI	1.9–3.7 mmHg	NA

Subgroup analysis paired *t*-test analyses of transcutaneous carbon dioxide (tcPCO_2_) accuracy. Sample size (*n*), mean values, mean of differences, and 95% confidence interval (CI) was reported for the difference between arterial carbon dioxide tension (P_a_CO_2_) and tcPCO_2_ values at 0, 60, and 120 s after arterial blood gas (ABG) sample collection for evening and morning ABG. AM tcPCO_2_(120) was unable by parametrically analyzed as it did not meet assumptions for the paired *t*-test.

**Table 3:** Sign test results

AM P_a_CO_2_–tcPCO_2_ (120)	(*n* = 29)
Negative differences	4
Positive differences	21
Ties	4
*p-*value (two-tailed)	=0.001

Results of the non-parametric sign test analysis of transcutaneous carbon dioxide (tcPCO_2_) electrode accuracy. The difference in morning arterial carbon dioxide tension (P_a_CO_2_) and tcPCO_2_ value 120 s after arterial blood gas sample collection was calculated, and the number of values >0, =0, and <0 determined. With four values <0, 21 values exceeding 0, and four values with no difference, a two-tailed *p*-value of .001 was determined for this analysis.

**Figure 1:**
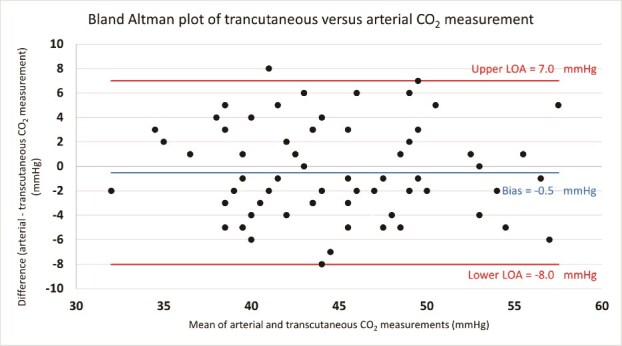
Bland–Altman plot for tcPCO_2_ measurements at 0 s. The above plot shows the Bland–Altman plot for assessing the accuracy of transcutaneous carbon dioxide (tcPCO_2_) represented by the difference between arterial carbon dioxide tension (P_a_CO_2_) and tcPCO_2_ vs. the average carbon dioxide value between techniques (P_a_CO_2_ + tCPCO_2/2_). A single outlier is noted to fall above the upper limit of the 95% confidence interval (>2 standard deviations above the mean bias of −0.5 mmHg).

A Bland–Altman plot was developed comparing the difference in P_a_CO_2_ and tcPCO_2_ at 0 s and the mean CO_2_ value obtained from these two modalities, with data shown in Figure 1. A mean bias of −0.5 mmHg is seen between the P_a_CO_2_ and tcPCO_2_ (0) values, with an upper limit of agreement of 7.0 mmHg and a lower limit of agreement of 8.0 mmHg. A single data point anomaly is plotted outside the limits of agreement (95% CI) in Figure 1. Linear regression of this plot revealed a standardized beta coefficient of −0.017 (*p* = .890) as shown in Table 4.

**Table 4:** Bland–Altman linear regression results

PaCO_2_–tcPCO_2_ (0)	*n* = 66
*M* ± *SD*	−0.5 ± 3.8 mmHg
95% CI	−8.0–7.0 mmHg
Linear regression	
Standardized beta coefficient	−0.017 (*p* = .890)
*R*^2^	0.000

Linear regression analysis of Bland–Altman plots (Figure 1) assessing transcutaneous carbon dioxide (tcPCO_2_) accuracy with variation in the magnitude of carbon dioxide. Sample size (*n*), mean values (*M*), standard deviation (*SD*), 95% confidence interval (CI) was reported for the difference between arterial carbon dioxide tension (P_a_CO_2_) and tcPCO_2_ values at 0 s, in addition to the standardized beta coefficient and R square value obtained from linear regression analysis.

## Discussion

In assessing the accuracy of CO_2_ levels determined by tcPCO_2_ compared to P_a_CO_2_ from ABG analysis, a significant difference was found for tcPCO_2_ measurements at all time points assessed. While statistically significant, the mean difference in tcPCO_2_ measurements in this study was found to be moderate and within clinically accepted limits, suggesting it is appropriate to utilize the SenTec tcPCO_2_ electrode in the PSG setting without the need for ABG to confirm arterial CO_2_ measurement.

tcPCO_2_ values taken at the point of ABG sampling were on average 2.7 and 2.2 mmHg less than the corresponding P_a_CO_2_ measurement for evening and morning measurements, respectively. These differences were statistically significant (*p* ≤ .001) and fell within the moderate difference (2–4.9 mmHg) category. Measurements were compared with separately for evening and morning ABG sampling to assess for sensor drift overtime, reported to be <0.5%/h for the SenTec tcPCO_2_ sensors [12]. The difference between evening and morning tcPCO_2_ values at all time points was minimal (<0.5 mmHg) suggesting no significant measurement drift over time, making them appropriate for temporal as well as point CO_2_ measurements.

The Bland–Altman plot demonstrated a random distribution of points with minimal bias related to CO_2_ level, with standardized beta coefficients close to 0. Despite a single point plotting outside the limit of agreement, points plotted randomly, and linear regression of the plot yielded a *p*-value of .890, suggesting no significant skew in the data distributions. Random distribution of values in both plots with no change in mean bias suggests no variation in accuracy of the SenTec tcPCO_2_ electrode with variation in the magnitude of arterial CO_2_.

Mixed results on tcPCO_2_ accuracy have been reported in the past [5, 14–17], with one systematic review performed across mixed clinical settings [10]. This study yielded a greater bias in CO_2_ measurement differences than that reported in the previous systematic review (2.7 and 2.2 vs. 0.1 mmHg), however revealed less data variation and a lower population limit of agreement, suggesting that the SenTec tcPCO_2_ is an appropriate tool to determine accurate CO_2_ levels in a PSG setting. Other influential variables identified by this systematic review included the type of tcPCO_2_ electrode and the electrode temperature; thus, a single type of tcPCO_2_ electrode (SenTec) and single temperature point (42°C) was utilized in this study.

The SenTec tcPCO_2_ electrodes have a reported response time of <75 s [12], thus measurements were also taken at 60 and 120 s after ABG sampling to assess the impact of CO_2_ stability on the effectiveness of tcPCO_2_ electrodes to accurately measure CO_2_. At 60 s, tcPCO_2_ values were 2.7 mmHg (evening) and 2.2 mmHg (morning) less than the P_a_CO_2_ value, while at 120 s the average tcPCO_2_ was 2.8 mmHg less than the average evening P_a_CO_2_ value. The tcPCO_2_ mean difference at 0, 60, and 120 s after the evening ABG varied between each other by just 0.1 mmHg whilst there was no variation in mean difference at 0 and 60 s for the morning ABG, suggesting no skew in accuracy based on the timing of measurements up to 120 s. At all time points discussed above, SenTec monitors yielded a moderate difference (2–4.9 mmHg) in CO_2_ level (using the upper 95% CI limit), were within acceptable clinical limits.

Some data points were missing for the morning tcPCO_2_(120) measurement due to conclusion of PSG and tcPCO_2_ monitoring before 120 s had elapsed after the morning ABG. As a result, <30 measurements were available for analysis and thus were unable to be analyzed via paired *t*-test due to the negative skew in the distribution of differences (P_a_CO_2_–tcPCO_2_(120)). A non-parametric sign test was used in place which showed a significant difference in median tcPCO_2_ (*p* = .001); however, the magnitude of this difference was unable to be determined.

An area not investigated due to missing database information resulting in a small sample size was the impact of tcPCO_2_ placement on the accuracy of the electrode, which has previously been reported to influence transcutaneous electrode accuracy [10]. tcPCO_2_ electrodes may be placed at different locations on the body if sleep laboratories lack guidelines for electrode placement or due to practicality restraints with the many wires and instruments set up during PSG to monitor EEG, respiratory, and abdominal muscle activity among other variables. As there is potential for different electrode placements to be utilized, knowledge of the effects on locational effects on electrode accuracy in the PSG setting are important to understand and may present an interesting opportunity for further research.

One limitation not accounted for during data analysis was the ABG conditions and potential impact of respiratory supports at the time of arterial sampling on CO_2_ levels at the time of sample collection. While most participants had their ABG samples collected whilst breathing room air immediately prior to/following the PSG study, a handful of participants had respiratory supports such as NIPPV or supplemental oxygen (at varying rates) operating whilst ABG samples were taken. This may have had the potential to alter CO_2_ levels detected by both ABG and the SenTec tcPCO_2_ electrode; however, the Bland–Altman plots produced from this dataset demonstrated no significant variation in accuracy with the magnitude of CO_2_ making this an unlikely influence over the determined tcPCO_2_ accuracy. Further investigations into the tcPCO_2_ clinical utility in the PSG population may consider alternative conditions as part of exclusion criteria to eliminate this potential limitation.

In patients undergoing PSG, SenTec tcPCO_2_ yielded lower CO_2_ measures than the current gold standard P_a_CO_2_ values determined by ABG at 0, 60, and 120 s following the evening ABGs (prior to PSG) and at 0 and 60 s following the morning ABG (after PSG). Whilst statistically significant differences were seen, these moderate differences with tcPCO_2_ monitoring remains within accepted clinical standards. This study demonstrated tcPCO_2_ as an appropriate avenue to pursue in determining CO_2_ levels in PSG cohorts, given the risk of infection, pain, hemorrhage in addition to the expertise and time of staff involved with arterial sampling for ABG analysis. Moderate differences were seen for each time point with only minor variations in the calculated mean difference; thus, it seems unlikely that instability and equilibration of arterial CO_2_ measurement in the SenTec electrode had any clinically significant impact on the accuracy of the tcPCO_2_ electrode, however, given the reduced sample size available for the morning tcPCO_2_(120) values further research in this area may be warranted to explore and confirm this finding.

## Data Availability

The data that supports the findings of this study is available upon written request to the corresponding author.

## References

[1] Comellini V, Pacilli AMG, Nava S. Benefits of non-invasive ventilation in acute hypercapnic respiratory failure. *Respirology.* 2019;24(4):308–317. 10.1111/resp.13469 30636373

[2] Gerdung CA, Adeleye A, Kirk VG. Noninvasive monitoring of CO_2_ during polysomnography: a review of the recent literature. *Curr Opin Pulm Med*. 2016;22(6):527–534. 10.1097/mcp.0000000000000320 27607154

[3] Zakynthinos S, Roussos C. Hypercapnic respiratory failure. *Respir Med*. 1993;87(6):409–411. 10.1016/0954-6111(93)90065-8 8210610

[4] Wilson M, Wang Z, Dobler C, et al. Noninvasive Positive Pressure Ventilation in the Home. Rockville, MD: Agency for Healthcare Research and Quality; 2019. http://www.ahrq.gov/clinic/epcix.htm. 32101390

[5] Bolliger D, Steiner LA, Kasper J, Aziz OA, Filipovic M, Seeberger MD. The accuracy of non-invasive carbon dioxide monitoring: a clinical evaluation of two transcutaneous systems. *Anaesthesia.* 2007;62(4):394–399. 10.1111/j.1365-2044.2007.04987.x 17381578

[6] Simmonds AK . Home mechanical ventilation: an overview. *Ann Am Thorac Soc.* 2016;13(11):2035–2044. 10.1513/AnnalsATS.201606-454FR 27560387

[7] Selim BJ, Wolfe L, Coleman JM, Dewan NA. Initiation of noninvasive ventilation for sleep related hypoventilation disorders: advanced modes and devices. *Chest*. 2018;153(1):251–265. 10.1016/j.chest.2017.06.036 28694199

[8] Worsnop C, Kay A, Pierce R, Kim Y, Trinder J. Activities of respiratory pump and upper airway muscles during sleep onset. *J Appl Physiol*. 1998;85:908–920. 10.1152/jappl.1998.85.3.908 9729564

[9] McNicholas WT . Impact of sleep in respiratory failure. *Eur Respir J*. 1997;10(4):920–933. 10.1183/09031936.97.10040920 9150336

[10] Conway A, Tipton E, Liu W-H, et al. Accuracy and precision of transcutaneous carbon dioxide monitoring: a systematic review and meta-analysis. *Thorax.* 2018;74(2):157–163. 10.1136/thoraxjnl-2017-211466 30209079

[11] Umeda A, Ishizaka M, Ikeda A, et al. Recent insights into the measurement of carbon dioxide concentrations for clinical practice in respiratory medicine. *Sensors.* 2021;21(16):5636–5655. 10.3390/s21165636 34451079 PMC8402333

[12] SenTec Digital Monitoring System . Noninvasive Ventilation and Oxygenation Monitoring. Instruction Manual for the SenTec Digital Monitoring System: Software Version SMB SW-V08.03. Therwill, Switzerland: Sentec. https://www.sentec.com/fileadmin/documents/Labeling/Instruction_Manuals/Archive/HB-005771-o-SDMS_InstructionManual_EN.pdf

[13] Severinghaus JW, Astrup PB. History of blood gas analysis. III. Carbon dioxide tension. *J Clin Monit Comput*. 1986;2(1):60–73. 10.1007/BF01619178 3086509

[14] Bendjelid K, Schutz N, Stotz M, Gerrard I, Suter PM, Romand J-A. Transcutaneous PCO_2_ monitoring in critically ill adults: clinical evaluation of a new sensor. *Crit Care Med*. 2005;33(10):2203–2206. 10.1097/01.CCM.0000181734.26070.26 16215371

[15] Lambert LL, Baldwin MB, Velasco Gonzalex C, Lowe GR, Randy WJ. Accuracy of transcutaneous CO_2_ values compared with arterial and capillary blood gases. *Respir Care*. 2018;63(7):907–912. 10.4187/respcare.05936 29739856

[16] Storre JH, Magnet FS, Dreher M, Windisch W. Transcutaneous monitoring as a replacement for arterial PCO_2_ monitoring during nocturnal non-invasive ventilation. *Respir Med*. 2011;105(1):143–150. 10.1016/j.rmed.2010.10.007 21030230

[17] Janssens J-P, Howarth-Frey C, Chevrolet J-C, Abajo B, Rochat T. Transcutaneous PCO_2_ to monitor noninvasive mechanical ventilation in adults: assessment of a new transcutaneous PCO_2_ device. *Chest*. 1997;113(3):768–773. 10.1378/chest.113.3.768 9515855

